# The potential of AI inspired approaches for patient monitoring in drug development for Huntington’s disease: capturing the digital exhaust for activities of daily living

**DOI:** 10.3389/fmmed.2026.1966010

**Published:** 2026-10-05

**Authors:** Amit Khanna, Graham Jones

**Affiliations:** 1 Neuroscience Development, Novartis AG, Basel, Switzerland; 2 Clinical Innovation, Novartis Pharmaceuticals, Cambridge, MA, United States; 3 Clinical and Translational Science Institute, Tufts University Medical Center, Boston, MA, United States

**Keywords:** artificial intelligence, digital twins, early detection, Huntington’s disease, passive monitoring

## Abstract

Diagnosis, assessment and monitoring of Huntington’s disease (HD) requires measurement of a complex array of motor, cognitive and psychiatric symptoms over sustained periods. However, the prodromal stages often span over a decade with symptoms presenting in a non-linear manner with short term fluctuations. This presents challenges for investigational drug development as treatment effects may become masked. To help address this issue, a number of digital health technology tools are being investigated for use in HD studies with increasing degrees of utility and sophistication. Herein we outline how remote passive monitoring technologies could be integrated into managed care of HD patients, and have the potential to inform algorithms and digital tools which could be used to improve our understanding of disease trajectories and improve early detection rates. Steps to implementation and the potential to derive learnings from adjacent technologies is presented, affording a key opportunity for next- generation approaches to patient monitoring. Measures derived from such technologies will also provide data to capture disease progression and treatment effects with high fidelity and granularity, and complement existing clinical research outcomes (clinROs) and patient reported outcomes (PRO’s). Given the ethical, economic, and societal burden presented by neurodegenerative diseases, such developments could have long term impact in managed healthcare.

## Introduction

Huntington’s disease (HD) is a neurodegenerative disorder caused by expanded CAG repeats in the IT15 HTT gene on chromosome 4p16.3. (Bates, 2005) HD is primarily genetic, results in cognitive decline and psychiatric issues coupled with uncontrolled movements, and is fatal, with post diagnosis median survival of just 15 years (Keum et al., 2016) in contrast to other neurodegenerative diseases (Tueth et al., 2024). HD is typically classified into premanifest and manifest phases with the extended prodromal phase often lasting a decade or more before clinical onset is formally diagnosed on the basis of unequivocal motor signs e.g., chorea. The hallmarks and symptoms of HD are typically heterogeneous, can vary over time in the same patient in a non-linear manner, and display short-term fluctuations. The difficulty in consistently detecting subtle early clinical manifestations poses challenges to therapeutic development, with clinical management requiring special care in the selection of assessment tools, which consider both physical and psychological burden on the patient (Exuzides et al., 2022).

Disease progression is assessed through motor, cognitive, and behavioral measures captured by established rating scales as the Unified Huntington’s Disease Rating Scale (UHDRS) and composite UHDRS (cUHDRS) a single overall progression measure, whose components include Total Functional Capacity (TFC), Total Motor Score (TMS), Symbol Digit Modalities Test (SDMT), and Stroop Word Reading (SWR) (Tabrizi et al., 2022). In addition the HD-ISS (Huntingtons disease Integrated Staging System) is developed for research and enabling evaluating disease modifying treatments early in the course of the disease. Despite widespread use, these (episodically deployed) scales suffer from natural limitations in capturing day-to-day variability of motor and other symptoms in clinical trials, potentially obscuring any treatment effects of investigational drugs, especially in early stages of the disease where capturing very subtle changes may provide insightful information (Carlozzi et al., 2025). This said, fluid biomarkers such as Cerebrospinal fluid (CSF) derived mHTT and neurofilament light chain (NfL) show correlations with symptoms, (Byrne et al., 2017) and new diagnostic assays including serum derived NfL (sNfL) (Byrne et al., 2017), and deregulated phosphatidylcholine metabolism (Yilmaz et al., 2025), coupled with metabolic and structural imaging methodologies (including CT/MRI, PET, and fMRI) show promise (Zeun et al., 2019). Given the urgency needed in drug development for HD, there is increasing interest in quantitative assessment of manifestations over sustained periods, with high-frequency monitoring of motor and cognitive function. Digital health technologies offer viable means of tracking these manifestations longitudinally at scale and a recent advance has been the development of the HD Digital Motor Score (HDDMS) for disease progression based on a smartphone platform (Giboin et al., 2025). This application adds to a growing stable of digital monitoring technologies developed for assessment of neurodegenerative diseases (Figures 1, 2) (Erickson et al., 2025). Evident benefits of using digital monitoring technologies include the potential to reduce friction and burden on the patient while at the same time driving to reduce variability in measurements, which can impact data quality. This balance is critical as high frequency data is desirous in order to detect subtle changes in symptoms, so optimal approaches must be geared towards increasing patient adherence and engagement through enhancing experience and or reducing burden (Figure 2) (Rosa et al., 2021). As digital technologies and data analytics capabilities evolve there is the realistic possibility for remote monitoring approaches to be developed including fully passive solutions, which may be capable of gathering vast amounts of relevant digital data to augment clinical data recorded during periodic assessment by attending clinicians. Once some disease modifying treatment options are available, and earlier diagnosis becomes feasible, such assessments would enable ‘shifting to the left’ and could serve HD patient populations very early in their disease. Moreover, by incorporating new AI methodologies in the process, there may be the potential for learnings uncovered to be deployed for next generation disease monitoring systems which will help identify progression in early stage patients with higher precision, and help optimize their care. This is particularly relevant when considering the prevalence and epidemiology of HD. As a Mendelian disorder, wherein children have a 50% probability of inheriting from parents with HD, its pathogenesis is molecular as opposed to environmental. Current estimates suggest it occurs with a frequency of 1 in 10,000 with higher disposition in persons of Caucasian descent (Yilmaz et al., 2025). In order to deploy accurate and reliable biomarkers able to track progression (or lack thereof) in clinical trials of potential disease modifying therapies new approaches are urgently needed, as the disease is fatal and symptoms complex. The possibilities of gathering passive digital measures longitudinally in the subjects natural environment offers the potential to augment clinician based assessment and monitoring in a meaningful capacity. Herein we outline potential strategies and highlight learnings from adjacent technologies which may help evolve this landscape in the near future.

**FIGURE 1:**
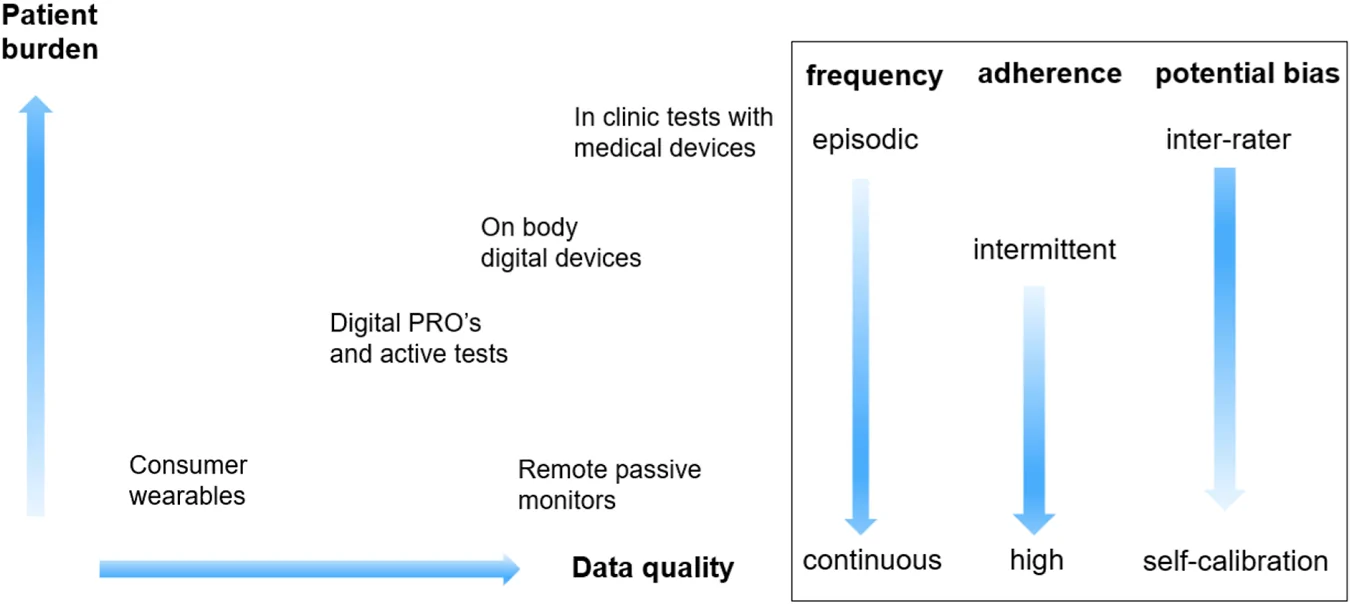
Friction levels for digital diagnostic and monitoring methods deployed in neurodegenerative disease studies.

**FIGURE 2:**
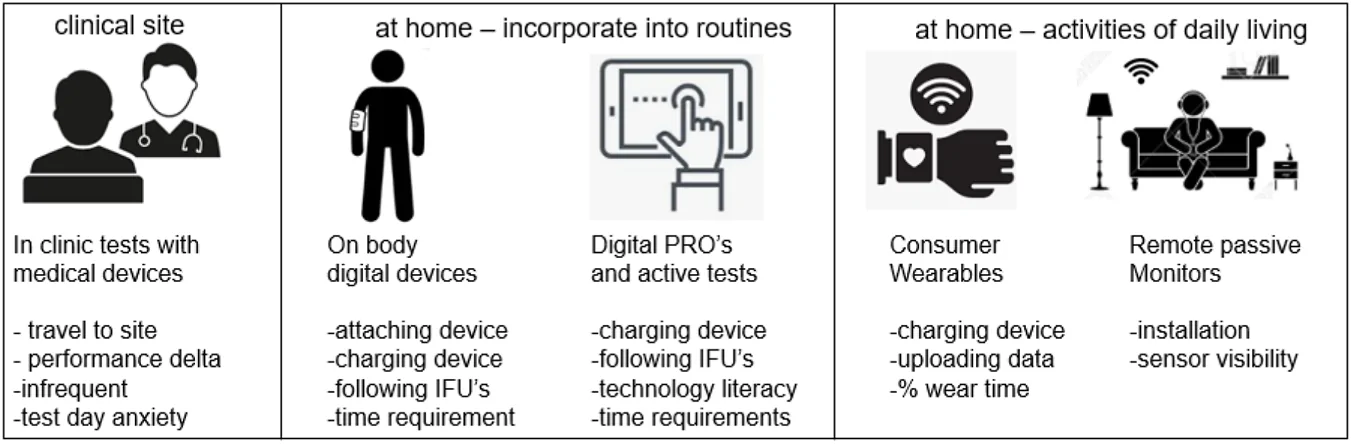
Friction and passivity level for diagnostic and monitoring methods in HD.

## Digital health technologies in HD

The deployment of digital tools for patient assessment in HD has benefitted from considerable research in related neurodegenerative and neuromuscular disease including multiple sclerosis, Parkinson’s disease and Alzheimer’s disease (DuBord et al., 2024). An evident benefit, accentuated during the COVID-19 pandemic has been the incorporation of PRO’s and other measures through telemedicine consults (Figure 2) (Khanna and Jones, 2021). A natural evolution of consumer-focused technology has seen deployment of various sensors and measurement devices which balance ease of use with technical precision and rigor. This includes on body devices such as truncal motion sensors, (Dinesh et al., 2020) gait measurement devices, (Tortelli et al., 2021) and tablet/smartphone based PRO and assessment tools a notable example being the HDDMS developed by Roche Diagnostics (Giboin et al., 2025; Waddell et al., 2021). Though accuracy relative to gold standard clinical approaches continues to improve, the degree of burden and limitations of scalability make widespread use challenging, and in many cases adherence lowers significantly over time, limiting the utility of the devices in the long term studies necessary (Giboin et al., 2025). For this reason recent developments have focused on consumer devices where activities of daily living can be tracked as part of the patients overall digital signature. The ultimate progression of such approaches could be envisioned in fully passive residential based Internet of Medical Things (IoMT) monitoring devices, where potentially rich data could be gathered with very high frequency (Mathur et al., 2024). Although yet to be examined in HD, one such example, using passive radio-frequency sensing has been deployed with some success in Parkinson’s disease and is able to capture features derived from motion, breathing, and sleep staging, correlating data with activities of daily living (Kabelac et al., 2019). As these data are potentially accessible with increased ease, it is important to consider how they best are incorporated into actionable insights for patients. In parallel, diagnosed patients will receive managed care through traditional channels and the data could be useful to present a real-world picture of activities of daily living which complement interval based assessment and care (Granström et al., 2020). There are other, digital and non-digital technologies which might also develop and could be considered as part of holistic means of achieving early diagnosis and tracking symptoms for those in care (Figure 3). These include the various features provisioned in wrist worn devices such as inertial measurement unit (IMU) sensors to assess movement anomalies and Propriospinal Myclonus (PSM) circuits which derive sleep pattern metrics, (Almujally et al., 2025) through to disease related idiosyncrasies such as signature keystroke patterns, (Liu et al., 2023) vocal anomalies detected from smartphones, (Perez et al., 2018), computers, or even voice assistant use. In some cases hallmark web and social media activities can correlate with disease symptoms (e.g., apathy, depression, anxiety) and could be useful components of health related metadata (Mansoor and Ansari, 2024). Modern smartphones and tablets also possess sophisticated microphone arrays, and cardinal acoustic signatures may be detected from daily interactions with the devices (Rowe et al., 2022). There are also a number of emerging opportunities for biomarker development using non-invasive methods. Among these are salivary based biomarkers of Huntingtin protein (Corey-Bloom et al., 2018) and interleukin-6 (IL-6), (Corey-Bloom et al., 2020) and analytes present in tears (Gijs et al., 2024), both of which have potential to be incorporated in to consumer-based assay kits, as popularized by flow-based COVID-19 assays. Finally, ocular analysis either through monitoring saccades using digital cameras, (Turner et al., 2011), or using benchtop instrumentation such as Optical Coherence Tomography (OCT) to detect retinal biomarkers has evident potential for early diagnosis (Gouravani et al., 2024). One might even envision non-traditional service providers such as optometrists and dentists providing such screenings and raising awareness for the disease and early diagnosis (Khanna et al., 2025). Although many of these potential diagnostics will take considerable effort to develop into clinically validated assys, learnings from other indications including Alzheimer’s and Parkinson’s disease offer insights and optimism that advances will follow (Mathur et al., 2024; Khanna et al., 2025).

**FIGURE 3:**
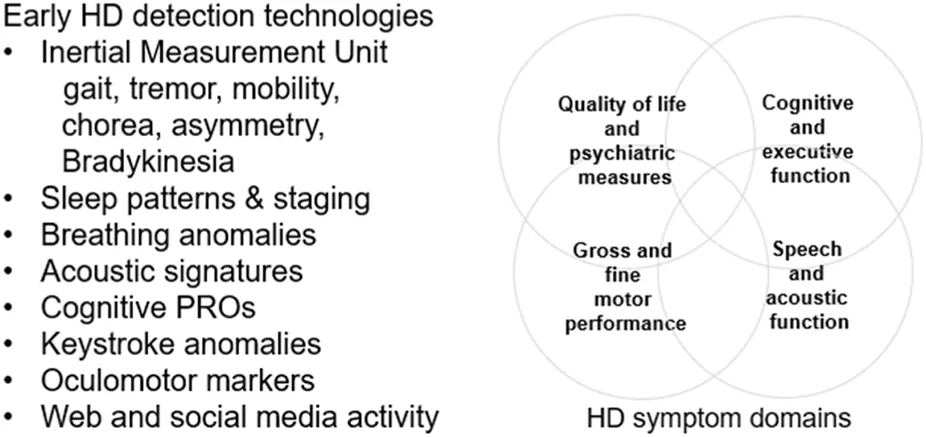
HD disease manifestation and symptom mapping and available DTH's.

## Integrating into daily living–the opportunity

The potential for digital sensor data to inspire development of monitoring tools for HD should not be understated. Given the complexities of the disease, its multiple symptoms, and its trajectory, it is incumbent on the research innovation community to play a role in collaborative development and advancement of next generation tools. Involving patient facing groups and research foundations in public-private partnerships between the technology and biopharmaceutical communities is a logical step and has already resulted in advances in other related diseases (Maxfield et al., 2017). Understanding and reflecting the needs of patients and their caregivers will be pivotal, and instrumental in any efforts to encourage sharing of potentially sensitive health data from personal devices. If approached appropriately, and building on recent learnings from digital health technologies, one could envision digital exhaust components playing a key role in development of composite measures for disease diagnosis and progression. By combining health tracking wearable data, web based activities, and sensors deployed in the home, this would present the opportunity for accumulation of large scale data to help drive research and encourage data sharing among those involved in drug development as has been observed in other diseases (Figure 4) (Khanna et al., 2024). These three components could help address the major domains associated with HD and its symptoms including:

**FIGURE 4:**
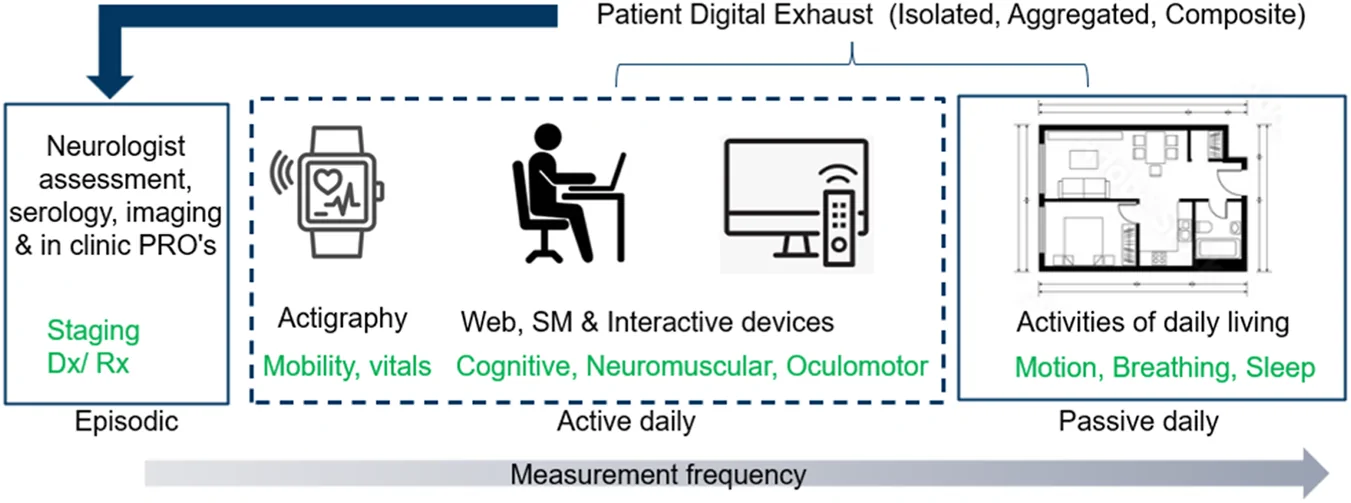
Digital exhaust components based on activites of daily living and IoMT home clinic approach to HD.

### Gross and fine motor performance

- Utilizing activity tracking wearables (gross motor skill, activity) in concert with IoMT based monitoring, active tests (keyboard, ocular) and passive monitoring (motion, keystroke, breathing).

### Cognitive function

- Motor function, executive function, memory recall, processing speed through gamified challenges on digital devices.

### Quality of life and psychiatric measures

- Behavioral assessment, apathy index, anxiety scoring through combination of web activity, sleep related measures, activity profiles and social media interaction.

### Speech and acoustic function

- Altered patterns, prosody, intonation, phonation and disease related features (e.g., pitch frequency, jitter, shimmer) through computer and smartphone interactions.

These data, in isolated, aggregate or composite form would offer a potentially vital component of the patients health record (Figure 4) and accurately represent elements of daily living, in the same manner that on board diagnostics in automobiles or flight data recorders in aircraft record data between scheduled service intervals. Many of these data might be captured passively and continually. For example, recent studies on smartphone use suggest that several psychopathologic behavioral markers including internalization, detachment, disinhibition, antagonism, and thought disorder may be derived from analysis of data from smartphone Global Positioning System (GPS) and IMU sensors when coupled with device activity logs and battery status (Ringwald et al., 2025). Combining such data with activities of daily living could help establish a highly relevant portrayal of the patients state of mind and wellbeing (Figure 4). Another opportunity is through smart residential video enabled devices such as computers and television monitors where symptom assessment could be enabled e.g., through gamification prompts, content-rich informational channels, and two way communications with health providers and social groups as necessary. The large scales offered in the graphic-user interface in these devices may help overcome inherent limitations of smart phones and tablets, providing a rich source of content and potentially higher user experience and engagement levels.

Bringing together technology developers, patient advocate groups and clinical trial sponsors to achieve the goals articulated will require careful coordination. Fortunately, organizations such as the Critical Path Institute have considerable experience in this area and have made notable inroads for the deployment of new digital approaches in related diseases such as Parkinson’s (Stephenson et al., 2023). There may be additional possibilities which could benefit from technologies not currently associated with healthcare. For example, in the area of home monitoring and security, a number of technologies are currently used to detect motion, room occupancy and even fall related events (Figure 5). These include radio frequency systems, (Zhang et al., 2020; Shaban et al., 2010) infra-red based motion detection (Suzuki et al., 2006), ultrasonic reflection technology (Liu et al., 2015), and mm RADAR monitors (Akbari et al., 2023; Saho et al., 2020; Hadjipanayi et al., 2024; Tewari et al., 2024). Additionally, accident avoidance systems such as echolocation could be adapted for in-home use to guide persons with motion and coordination impairments (Sohl-Dic et al., 2015). Many of these platforms are ideally suited for home health monitoring, with the waveforms able to penetrate home structural features, function nocturnally, and tolerate challenging environments, e.g., steam, humidity (Lai et al., 2015). A collaboration between such product developers and the health technology industry could be a fertile ground for development, and if curated algorithms tailored to the idiosyncrasies of HD were made available (possibly staged based on disease classifiers) it could help accelerate the development cycle. Such advances could have benefits and application in other neurodegenerative and neuromuscular diseases, and it is easy to see how a marriage of smart home technologies based on security and mobility could create business value for potential investors (Frishammar et al., 2023). One possibility could be to facilitate demonstration projects in assisted living facilities which cater for those with HD and related diseases, jump starting the commoditization of technologies which later become available to consumers (Liyakathunisa et al., 2021). Clearly, many possibilities exist, and it is incumbent on us to help foster the necessary collaborations and partnerships.

**FIGURE 5:**
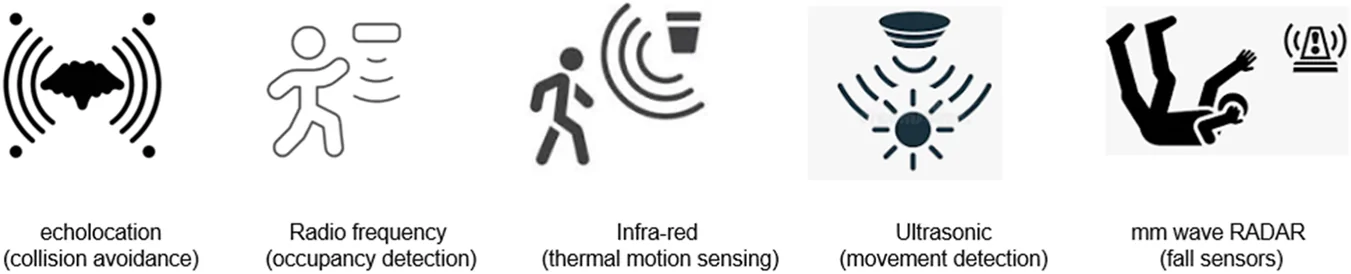
Potential consumer safety and home sensor technologies for passive monitoring in HD.

## Methods

For the identification, assessment and prioritization of the technologies described herein a comprehensive literature search was conducted using commerical engines (SciFinder, Google Scholar) with emphasis on the period 2016-present. Cited exemplars were selected on the basis of relevance to HD, impact factor and potential applicability in clinical development.

## Conclusions and recommendations

Digital health technologies and AI methodologies are advancing rapidly and having a marked impact on our understanding of neurodegenerative diseases including HD. With appropriate development these technologies could advance to become central pillars in clinical trials and drug development and provide means to enhance critical care of patients. Already through apps such as the remotely administered Roche HD Digital Motor Scoring system, we have a natural extension of physician/clinic based episodic patient monitoring, (Giboin et al., 2025) and integrating digital derived activities of daily living from the home environment represents a logical evolution towards holistic patient care. Such may also serve to help inform and enhance clinical endpoint development, where existing scales and measures when used alone suffer from limitations in early stage disease due to limited sensitivity to capture meaningful change in a reasonable time span and owing to the disease heterogeneity (Tabrizi et al., 2022).

Collectively this necessitates extended observational periods and larger clinical trial cohorts, adding to both cost and timelines, which provides added impetus to the approaches proposed herein. Approaches and measures described herein also have the potential to capture disease progression and treatment effects with high fidelity and granularity, complementing existing clinRO’s and PRO’s. In all of the above, a critical component will be to develop standards for data security, interoperability and harmonization to allow optimal use of the captured data (Khanna et al., 2024). Established standards such as Fast Healthcare Interoperability Resources (FHIR) and Health Level Seven (HL7) have addressed many issues but integration with patient electronic health record systems will require careful alignment given variations across healthcare systems globally, along with region specific data protection standards e.g., General Data Protection Regulation (GDPR), Health Insurance Portability and Accountability Act (HIPPA).

We issue this call to arms for technology developers to join in collaborative efforts for instrument and device design and urge the readers of this journal to consider avenues for implementation. Potential next steps could involve:

1. Convening dedicated dialog between pharmaceutical industry delegates, HD experts and research foundations (such as CHDI), professional medical societies, and digital health technology developers on digital concepts of interest.
2. Engaging integrators such as the Critical Path Institute to begin dialog with health authorities regarding the design of composite measures and endpoints.
3. Incentivizing data sharing and federated learning consortia through grant funding opportunities e.g., the Innovative Health Initiative, the US National Institutes of Health (NIH), and the Foundation for the NIH.
4. Reimagining endpoints and scales such as the cUHDRS to benefit from advances in digital monitoring technologies.
5. Convening working groups to propose and design relevant architecures, frameworks and interoperability standards to allow data capture, storage, sharing and interrogation.

We envision highlighting this opportunity at an upcoming professional society meeting and engage in active dialog on these important topics.
